# Role of Human Galectins in Inflammation and Cancers Associated with Endometriosis

**DOI:** 10.3390/biom10020230

**Published:** 2020-02-04

**Authors:** Brooke V. Hisrich, R. Brant Young, Alison M. Sansone, Zachary Bowens, Lisa J. Green, Bruce A. Lessey, Anna V. Blenda

**Affiliations:** 1 Department of Biomedical Sciences, University of South Carolina School of Medicine Greenville, Greenville, SC 29605, USA; bhisrich@email.sc.edu (B.V.H.); youngrb@email.sc.edu (R.B.Y.); asansone@email.sc.edu (A.M.S.); 2 Department of Obstetrics and Gynecology, Prisma Health, Division of Reproductive Endocrinology and Infertility, Greenville, SC 29605, USA; zcbowens@gmail.com (Z.B.); lisa.green2@prismahealth.org (L.J.G.); blessey@wakehealth.edu (B.A.L.); 3 Department of Obstetrics and Gynecology, Wake Forest School of Medicine, Winston-Salem, NC 27157, USA

**Keywords:** galectin, pathophysiology, endometriosis, inflammation, cancer

## Abstract

Galectins are a family of β-galactoside-binding proteins that contribute to multiple cellular functions, including immune surveillance and apoptosis. Human galectins are also important regulators of inflammation, making them a research target for various inflammatory diseases and tumorigenesis associated with pro-inflammatory conditions. This review focuses on the involvement of human galectins in modulation of inflammation and in the pathophysiology of endometriosis and endometriosis-associated neoplasms. Endometriosis is a chronic inflammatory disease with unknown etiology. Galectins-1, -3 and -9 were found to be overexpressed in ectopic and eutopic endometrium of females with endometriosis compared to those without endometriosis. These findings suggest galectins’ role in the progression on endometriotic lesions and their potential use as diagnostic biomarkers and/or targets for therapeutic approaches. Galectins-1, -3, and -9 have also been implicated in the development of endometriosis-associated neoplasms. Furthermore, galectin-3 has been shown to interact with KRAS protein and contribute to cellular growth, proliferation, inflammation, and the uptake of nutrients in endometriotic lesions and may be involved in the maintenance and propagation of endometriosis. These galectins have been shown to be upregulated in certain forms of cervical, ovarian, endometrial, and colon cancer associated with endometriosis and have become a potential target for anti-cancer therapies.

## 1. Introduction

Galectins are glycan binding proteins that bind specifically to β-galactosides. They are found in virtually all organisms, can be intracellular or extracellular, and are thought to have multiple functions [1]. Galectins are known to contribute to cell-to-cell interactions, cell-extracellular matrix (ECM) interactions, cell surface signaling, modulation of intracellular processes such as pre-mRNA splicing, and regulation of apoptosis; they also play a role in cell adhesion, migration, angiogenesis and invasion, and inflammation [2]. There are 12 known human galectins, each with their own specific tissue distribution [1]. Galectins-1, -3 and -9 have been shown to be linked to the development of neoplasms, including gynecological cancers, and may also contribute to the development and progression of endometriosis and ectopic endometrial growth [2].

Endometriosis is an inflammatory disease that primarily affects women during their reproductive years [3]. The disease is characterized by the presence of endometrial glands and stroma outside the uterus that is often associated with severe pelvic pain and infertility [3]. Endometriosis is a benign, chronic disease that affects nearly 10% of the female population [3]. Although the etiology and pathogenesis of endometriosis are largely unknown, certain galectins are upregulated in endometriosis. Overexpression of galectins-1, -3 and -9 was detected in female reproductive tissues, such as endometrium stroma and decidua, suggesting their role in the progression and maintenance of endometriotic lesions [3]. 

Due to the involvement of ectopic endometrial glands and stroma in endometriosis, it is crucial to understand the effects of monthly menstrual changes that occur to the endometrium with each cycle. In addition to the established inflammatory nature of endometriosis [3], regular monthly menstrual cycles also exhibit signs of acute inflammation [4,5]. Regular premenstrual symptoms such as irritability, abdominal cramps, water retention, weight gain, and depression are positively correlated with elevated high-sensitivity C-reactive protein (hs-CRP) levels, a known marker of acute inflammation [4,5]. There seems to be a linear association between the levels of CRP, and thus levels of inflammation, with levels of premenstrual symptoms [5]. While those results present CRP as a marker of the acute inflammation of monthly menstruation, our review focuses on the possible role of galectins in acute inflammation associated with monthly menstruation as well as endometriosis. Both galectins-1 and -3 have been implicated in the initiation and modulation of inflammation [1], which makes them prime markers for the inflammation in endometriosis and will be examined further in this review. 

Additionally, endometriosis and chronic inflammation of the pelvis are also associated with certain forms of cancer. Under normal physiological conditions, galectins help to regulate the cell cycle and prevent tumor formation. However, when galectin function is affected, an environment favorable for cancer development and metastasis is created. Galectin expression also modulates the immune response. Abnormal galectin expression can reduce T cell responsiveness to neoplastic cells and increase the risk of tumor development and metastasis [6]. Due to these associations, recent studies have investigated the usefulness of galectin inhibitors in treating certain forms of cancer associated with increased galectin levels. This review will outline the roles of galectins in the development of gynecological cancers and discuss ways in which galectin inhibitors can be used in cancer treatment. 

## 2. Role of Human Galectins in the Pathophysiology of Endometriosis 

Galectins-1, -3, -9, and -15 are expressed in both human endometrium and decidua, and galectin-1 is also expressed in endometrial stroma [3]. Galectins’ role in the regulation of endometrial immune and inflammatory responses may be a contributing factor in endometriosis development and pathogenesis. Expression of specific galectins was studied in women with and without endometriosis to explore galectins as both potential diagnostic biomarkers and targets for endometriosis therapy.

### 2.1. Overexpression of Galectin-1 in Endometriosis 

Galectin-1, which is expressed in the endometrium, plays a critical role in implantation and trophoblast invasion [3]. Its expression in both endometriotic lesions and normal eutopic endometrium was examined during proliferative and secretory phases of the menstrual cycle. It was demonstrated that galectin-1 is overexpressed in endometriotic lesions compared to eutopic endometrium of women with endometriosis and is more abundantly expressed in eutopic endometrium of affected women compared to eutopic endometrium of women without endometriosis. Galectin-1 interferes with inflammatory responses in two different ways because it can act as both a pro-inflammatory and anti-inflammatory cytokine. These results suggest that galectin-1 may play an important role in the pathology and progression of endometriosis [3].

### 2.2. Galectin-1 Promotion of Angiogenesis in Endometrial Growth Outside the Uterus 

Endometriosis is suspected to involve the chronic dysregulation of both inflammatory and vascular signaling [3]. Galectin-1 is selectively expressed in both human endometrial endothelial cells and stromal cells in ectopic and eutopic endometrium. An experimental model with both wild type (WT) and galectin-1-deficient mice was used, and endometriosis was induced in both the WT and galectin-1-deficient mice [3]. The results indicated that galectin-1 facilitated the formation of vascular networks in endometriotic lesions, leading to further ectopic growth independent of vascular endothelial growth factor (VEGF) in the WT mice [3]. Moreover, no further angiogenesis was noted in the galectin-1-deficient mice, indicating the important role galectin-1 may play in angiogenesis and thus further proliferation of ectopic endometriotic lesions [3]. Monoclonal antibodies selectively targeting galectin-1 were found to significantly reduce the size of the lesion and restrict the vascularized area. The inhibition of angiogenesis using a monoclonal antibody targeting galectin-1 could potentially slow the progression of endometriotic lesions and ectopic growth that progresses endometriosis [3]. Therefore, galectin-1 may be one of the next targets of novel therapeutic approaches for the treatment of endometriosis.

### 2.3. Galectin-3 Overexpression in Endometriosis 

Galectin-3 plays an important role in cell adhesion, migration, angiogenesis and cellular invasion [7]. Expression of galectin-3 was investigated using immunohistochemistry (IHC) in both patients with endometriosis and negative controls. It was found that both in the proliferative and secretory phases of the menstrual cycle, nuclear and membranous galectin-3 expression was highest in women with endometriosis, second highest in eutopic endometrium of women with endometriosis and lowest in eutopic endometrium of women without endometriosis [7]. This study suggests that galectin-3 also may play a role in the development and/or progression of endometriosis [7]. 

Importance of galectin-3 overexpression in the development of endometriotic lesions and the effect of recombinant galectin-3 carbohydrate recognition domain Gal3C in an experimental endometriosis treatment were demonstrated [8]. In addition, pentamer galectin-3 can crosslink glycosylated ligands to form a lattice on the cell surface that regulates receptor kinase signaling and the function of different membrane receptors [9]. Galectin-3 can be proteolytically cleaved by metalloproteinases (MMPs) to form a truncated recombinant galectin-3 carbohydrate recognition domain Gal3C, which binds to glycoconjugates more tightly and has been indicated to play an opposing role to galectin-3 [9]. Moreover, Gal3C has the same ligand binding site as galectin-3 and it may competitively bind to the same ligands, disrupting galectin-3 dependent signaling cascades [9]. 

Gal3C has been investigated for its potential anti-tumor and anti-angiogenesis properties [8]. It was found that both galectin-3 deficiency and Gal3C presence impaired endometriosis development, and there was a significant reduction in both the lesion implantation and size. With galectin-3 inhibition or absence, VEGF, an indicator of angiogenesis, as well as transforming growth factor beta (TGF-β) and cyclooxygenase-2 (COX-2), both indicators of inflammation, were also downregulated [8]. Under normal conditions, VEGF, TGF-β, and COX-2 are the likely promoters of endometriosis development and progression due to their activation of further angiogenesis and inflammatory responses, respectively. This study indicates a possibly significant role of galectin-3 in endometriosis development and highlights the potential therapeutic approach of galectin-3 inhibition in the treatment of endometriosis [8]. 

Lastly, although the data support galectin-3 inhibition in the reduction of endometriotic lesions, it should be considered that galectin-3-dependent carbohydrate binding is critical for the initiation of multiple signaling cascades involved in the promotion of cell-to-cell adhesion and inflammatory responses [8]. Complete inhibition of galectin-3 or the delivery of Gal3C may lead to other downstream effects impacting or disrupting signaling cascades independent of endometriosis development.

### 2.4. Galectin-9: Novel Endometrial Marker for Mid-Late Secretory Phase 

Expression and regulation of galectin-9 was investigated in human ectopic and eutopic endometrium [10]. Galectin-9 mRNA and protein from endometrial biopsies of various stages of the menstrual cycle including proliferative and secretory phases were analyzed. Galectin-9 mRNA expression was quite low in the proliferative phase and the early secretory phase but showed a drastic increase in expression during mid and late secretory phases in the endometrial cells, but not the stromal cells. The regulation of galectin-9 activity by estradiol, progesterone, epidermal growth factor, and interferon-γ was not detected. Based on the results of this study, galectin-9 could potentially be used as a novel endometrial marker to mid- and late secretory phases [10]. It was also hypothesized that galectin-9 might be a strong indicator of endometrial receptivity prior to implantation in normal, non-diseased endometrium [11].

### 2.5. Galectin-9: Noninvasive Biomarker for Detection of Endometriosis and Gynecological Disorders

Potential use of serum galectin-9 as a biomarker for the detection of endometriosis and other gynecological disorders is under consideration [11]. The potential of soluble galectin-9 as a diagnostic biomarker is particularly significant because it can be detected without laparoscopic diagnostic surgery, a standard way endometriosis and other gynecologic disorders are currently being diagnosed. A collection of tissue biopsies, peritoneal cells, and native peripheral blood from both women with and without endometriosis was collected [11]. There were significantly elevated levels of soluble galectin-9 in both minimal/mild stages of endometriosis (stages I-II) and in moderate/severe stages of endometriosis (stages III-IV) in comparison to the negative controls. 

The study determined that galectin-9 ELISA had a sensitivity of 94% and a specificity of 93.75% for detection of endometriosis, which indicates soluble galectin-9 may be a better diagnostic biomarker for endometriosis compared to cancer antigen 125 (CA 125), interleukin-6 (Il-6), tumor necrosis factor alpha (TNFa) and VEGF [11]. Currently, one of the most widely used biomarkers for endometriosis is CA 125, a glycoprotein that is used as a noninvasive biomarker for epithelial ovarian cancer. CA 125 expression is also upregulated in late stages of endometriosis but proves to be inadequate for detection of early stages of endometriosis [11]. In addition to the elevated levels of galectin-9 in women with endometriosis, increased serum galectin-9 levels were also found in women with varying benign gynecologic disorders, pelvic pain, and infertility [11]. 

## 3. Human Galectins and Their Involvement in Inflammation

### 3.1. Galectin Functions in the Endometrium 

Expression of galectins in the endometrium was investigated during both the proliferative and secretory phases of the uterine cycle, as well as during implantation of an embryo. Pro-inflammatory galectins-1 and -3 were seen to be strongly elevated during the proliferative phase [12]. Galectin-1 was seen to be significantly expressed in both the proliferative and secretory phases at similar rates, and mRNA expression was the highest in the stromal cells of the endometrium [13]. Galectin-3, on the other hand, was low in expression during the proliferative phase with a 3-fold increase during the secretory phase. Galectin-3 expression was rather low in the stromal cells but strongly shown in the glandular epithelial cells which correlates to its rise during the secretory phase when these glands are most active [12]. 

On the other side, galectin-7 has been associated with the anti-inflammatory recovery during menstruation. Endometrial galectin-7 accumulates in menstrual fluid and is released from endometrial stromal cells during the late secretory phase of menstruation. Galectin-7 in the accumulating menstrual fluid has been shown to play a role in the production of extracellular matrix factors which allows for re-epithelialization and tissue repair towards the end of menstruation leading to bleeding termination [14]. While most research on galectin-7 has been linked to skin epithelia, these are early connections to its role in the endometrium with recovery from menstruation. 

The balance between the pro- and anti-inflammatory effects of galectins highlights the varied roles galectins play around the body and specifically in the endometrium as well. 

### 3.2. Specific Roles of Galectins-1 and -3 in General Inflammation

Further investigation of the pro-inflammatory roles of galectin-1 and -3, not specific to endometrium, showed that galectins are produced by epithelium and activate macrophages and endothelial cells during inflammation as well as bacterial infection [15]. As was described earlier, menstruation results in a rise in CRP levels, indicating acute inflammation that could trigger this galectin release [4]. The release of galectins-1 and -3 functions in several ways to initiate an inflammatory response. These functions include facilitating the binding of neutrophils to the endothelium, trafficking of neutrophils through the extracellular matrix by binding to laminins and fibronectin, as well as acting as chemotactic agents towards the site of inflammation [15]. Once neutrophils have been trafficked to the scene, galectins-1 and -3 then contribute to the respiratory burst that allows neutrophils to destroy cellular contents or invading pathogens [15]. While previously the role of galectins-1 and -3 were discussed to be elevated during the uterine cycle, this investigation helps to connect the phases of the uterine cycle to actual inflammatory damage at the cellular level. 

### 3.3. Role of Galectin-1 in the Regulation of Inflammation

While galectin-1 plays an important role in initiating inflammatory responses, it has also been shown to be an important regulator of inflammation. Galectin-1 is overexpressed in the regulatory CD4+ CD25+ regulatory T cells, which can modulate the response of the adaptive immune system upon activation [16]. The role of galectin-1 in extracellular trafficking described above represents an innate immune response, though when employing the adaptive response, galectin-1 appears to regulate the level of response. Expanding on the role of galectin-1 as a regulator of the adaptive immune system, galectin-1 was shown to inhibit MHC Class II expression induced by interferon gamma (IFN-γ) and limit the MHC Class II antigen presentation capability [17]. These are both critical roles to initiating an adaptive response by the immune system, and they are limited by galectin-1. 

### 3.4. Importance of Galectin-3 in Long-Term Inflammatory Processes 

Galectin-3 has been shown to have a unique role in the transition from acute to chronic inflammation over time [18]. With continued tissue injury or damage, galectin-3 has been implied in facilitating the walling off of chronic inflammation that comes with fibrogenesis and scarring to isolate the damage from the surrounding healthy tissue. As the inflammatory processes described above become more and more frequent, galectin-3 appears to play a role in isolating the damage and transitioning the response to chronic inflammation [18]. Since menstruation occurs every month, the repeated inflammation and associated increase in galectin-3 production during the secretory phase could contribute to chronic inflammation associated with the menstrual cycle. 

### 3.5. Roles of Galectin-3 in Endometriosis-Related Inflammation 

Galectin-3 has been shown to propagate the development of endometriotic lesions via angiogenic factors [8]. One marker of endometriosis is pelvic inflammation. The exact mechanism of this inflammation has not yet been elucidated. However, galectin-3 and ST2, a member of the interleukin (IL)-1 receptor family, had significantly higher concentrations in the peritoneal fluid (PF) of women with endometriosis compared to controls without endometriosis. The difference in protein concentrations was found to be proportional to the stage of endometriosis [8]. 

Galectin-3 has been shown to modulate inflammation through T-cell apoptosis via caspase-9 activation [19,20]. In addition, the export of phosphorylated galectin-3 from the nucleus into the cytoplasm has been shown to exhibit anti-apoptotic activity by preventing both the release of cytochrome-c and caspase-3 activation [21,22,23]. The pro- and anti-apoptotic properties of galectin-3 may serve as a possible explanation for the increase in galectin-3 concentration in the PF of women with endometriosis. 

Furthermore, a drop in pH can be associated with inflammation, and the inflammatory agent may be a factor that influences the severity of the pH drop [24]. The activity of galectin-3 has been seen to be modulated by environmental pH [25], and even slight changes in pH modulates the structure and activity of galectin-3 [26]. These findings indicate that even slight variations in pH, such as those induced by inflammation, may contribute to the possible mechanism for the modulation of galectin-3 activity and thus influence its pro- and anti-inflammatory properties.

### 3.6. Immune and Inflammatory Consequences of Galectin-3 Deficiency

It is also important to investigate the consequences of galectin-3 deficiency. It was shown that galectin-3-deficient mice developed fewer instances of inflammatory cell infiltration, had decreased level of macrophages, developed lower nuclear factor kappa-light-chain-enhancer of activated B cells (NF-κΒ) inflammatory responses, and were more likely to experience macrophage apoptosis [27]. The phenotype of galectin-3-deficient mice suggests that galectin-3 is involved in the survival of inflammatory cells and promotes their ability to function in inflammatory responses [27]. 

### 3.7. Galectin-3 and KRAS Interactions in Endometriosis-Related Inflammation

The oncogene *KRAS* codes for the KRAS protein that is primarily involved in the mitogen-activated protein kinases (MAPK) and phosphoinositide-3 kinase (PI3K) pathways [28]. As such, KRAS serves as an important early component in cellular development and maturation. As a GTPase transmembrane protein, GTP-activated KRAS has been implicated in the development of endometriosis [29]. In both mouse and human models, it has been shown that activation of KRAS stimulates the development of endometriotic lesions [30]. Furthermore, KRAS has been shown to be a prevalent biomarker in the endometrium of women with endometriosis [31].

KRAS has been shown to interact with intracellular galectin-3. Activated KRAS recruits cytosolic proteins to the plasma membrane. The recruitment of specific cytosolic proteins depends upon the specificity of the binding ligand and indicates which cascade the KRAS protein will induce [32,33,34]. KRAS has been shown to recruit cytosolic galectin-3 upon binding of epidermal growth factor (EGF). The recruitment of galectin-3 has been implicated as a necessary component in the “stability of KRAS GTP-loading and regulation of signal output” [35]. However, how galectin-3 stabilizes the KRAS-GTP complex is not yet known [36]. 

Cytosolic galectin-3 was shown to directly interact with the KRAS-GTP complex to modulate the effects of EGF stimulatory factors such as cellular proliferation, anchorage-independent cellular growth, and inhibition of apoptosis in a dose-dependent fashion [37]. Furthermore, galectin-3 and KRAS have been associated with some epithelial cancers [38]. Activation of KRAS by galectin-3 in anchorage-independent cells has been shown to induce macropinocytosis [39]. These findings make galectin-3 a target of interest for investigation of the growth, proliferation, and inflammation that occurs in endometriotic lesions. 

## 4. Endometriosis-Associated Neoplasms 

Endometrial-associated cancers are characterized by endometrial tissue attached to or closely associated with the tumor, and the histology of the tumor must be consistent with an endometrial origin. The most common forms include clear cell and endometrioid ovarian cancer, though certain types of cervical and colorectal cancer have also been implicated [6]. 

**Figure 1:**
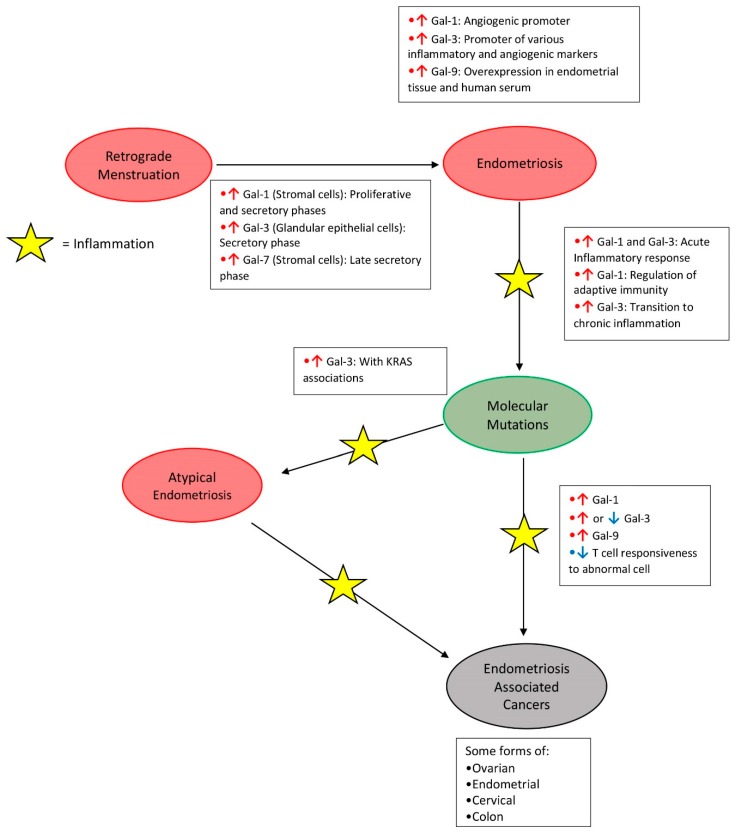
Modified model for potential development of endometriosis-associated cancers and changes occurring in the expression of human galectins. Red arrows indicate increased galectin expression, blue arrows indicate decreased galectin expression. Original model was proposed by Dawson et al. [40].

Additionally, endometriosis can be linked to both altered galectin expression and associated increased risk of certain forms of gynecological cancers [40]. Figure 1 describes a potential model of those associations. Chronic inflammation in the tissues invaded by endometriosis further promotes dysplasia. Inflammation leads to the increased activity of immune system, most notably in the form of altered gene expression of the complement components. Overexpression of complement has been linked to tumor growth through various mechanisms [40]. Endometriosis tissue also demonstrates a notable increase in somatic mutations of important tumor suppressor and oncogenes. These somatic mutations appear to be an important factor in the transformation of endometriosis into cancer. 

Upregulation of KRAS and phosphatase and tensin homolog (PTEN)-regulated pathways have been linked to an upregulation of the complement pathway, as seen in endometriosis. Mutations in tumor suppressor genes such as ARID1A and PIKA3CA have been found in patients with both endometriosis and an endometriosis-related cancer [41]. Galectins help to modulate the pathways controlled by many oncogenes and tumor suppressor genes. Abnormal levels of galectins can cause increased malfunction of these pathways [42]. The altered levels of galectin expression and increased mutation rate within endometriosis, along with its tendency to cause chronic inflammation, make it unstable and can lead to the ability to invade and metastasize [43].

The galectins that show the strongest association with human neoplasms related to endometriosis are galectins-1, -3, and -9. Each of these have been shown to be affected in a variety of tumor types, and their mechanism of action often varies between tumor types [42]. In cancer cells, intracellular galectins are more commonly associated with modulation of oncogenic signaling pathways and alterations in apoptosis and proliferation rates. Extracellular galectins and their receptors are associated with tumor cell adhesion and transportation [42]. Additionally, all galectins implicated in cancers have been linked to immunosuppression and immune escape by tumor cells.

### 4.1. Role of Galectin-1 in Tumorigenesis

In endometrial cancer, galectin-1 levels are significantly increased compared to normal tissue, but there is conflicting evidence as to the prognostic value of galectin-1 levels. Galectin-1 levels were reported by one study to increase as tumor grade increases, but other studies have not demonstrated such a relationship [43]. Galectin-1 is also overexpressed in endometriosis-associated ovarian cancer (EAOC). It is particularly important when galectin-1 is high within stromal cells, where it is linked to poor outcomes and can be utilized as an independent prognostic tool [43]. Galectin-1 binds numerous cell surface and ECM receptors, including a variety of integrins and laminins. These interactions are important for survival signaling and cellular adhesion. In cancer, these interactions can promote tumor cell survival and transportation [44].

### 4.2. Role of Galectin-3 in Tumorigenesis

Squamous cell carcinoma of the cervix has been linked to endometriosis [42]. Liu et al. proposed a mechanism of tumor invasion involving galectin-3 interactions with VEGF. They showed that galectin-3 propagates the VEGF pathway, leading to increased angiogenesis and expansion of the tumor [45]. In these tumors, low levels of galectin-3 are indicative of a more aggressive and invasive tumor, while normal or even high levels of galectin-3 were associated with less invasive tumors. This generalization does not hold true for all tumors, however. 

Clear cell carcinoma is a common form of EAOC. Galectin-3 expression in clear cell carcinoma is associated with higher migratory and invasive capability [43]. In these cells, galectin-3 is associated with the regulation of NF-κΒ, an important player in the regulation of cell survival and proliferation. Expression of galectin-3 within ovarian cancer cells has been shown to upregulate the NF-κΒ pathway [43]. Galectin-3 expression in EAOC is also an indicator of higher stage tumors, making it a possible prognostic marker for EAOC.

In many endometrial cancers, galectin-3 expression is reduced, and low levels of galectin-3 may be involved in the transformation of normal endometrium into carcinoma. However, in certain tumors, galectin-3 levels are higher than in normal tissues [43]. These tumors with increased expression of galectin-3 are often stage III or IV and indicate that increased galectin-3 expression may be associated with more advanced endometrial disease [43]. Galectin-3 is often low in slow-growing tumors, but high levels of galectin-3 indicate more aggressive forms of cancer with higher rates of metastasis and poor outcomes [43].

The variable levels of expression found in different stages and types of gynecological cancer make galectin-3 an interesting target for prognostic testing. Additionally, evidence suggests that galectin-3 may be linked to higher rates of resistance to several common chemotherapeutic agents such as paclitaxel and carboplatin [43]. Evidence suggests that galectins can increase the expression of the multidrug resistance protein (MDR) [42]. MDR enables the cell to pump chemotherapy out of the cell, reducing efficacy. This would suggest that galectin-3 may not only be valuable in staging a tumor, but also in evaluating its susceptibility to chemotherapy.

### 4.3. Role of Galectin-9 and Immunomodulation in Tumorigenesis

Though galectin-1 and galectin-3 are also immunomodulators, galectin-9 is widely considered to be the strongest immune modulator related to cancer [42]. One mechanism in which galectins modulate cell survival and affect immune function is through the ability to bind and trigger apoptosis. Galectins bind anti-tumor T cells and induce their apoptosis [42]. When galectins are upregulated in tumor cells, it can help tumor cells to evade the immune response against them. Furthermore, it has been shown that galectins can increase the activity of T regulatory cells, increasing the immune tolerance for tumor cells. The effect of galectin-9 on tumor cells is dependent upon its ligand, T-cell immunoglobulin and mucin-domain containing-3 (Tim-3). Galectin-9 binding the Tim-3 receptor on T helper cells is associated with apoptosis of T cells, increased immune tolerance to tumor cells, higher rates of metastasis, and poor prognosis for patients. In contrast, when galectin-3 binds carbohydrates on the surface of tumor cells, it induces apoptosis of these cells and increases the immune response against the tumor cells [42].

## 5. Therapeutic Approaches

The ability of galectins to modulate tumor growth and expansion suggested them as targets for new treatment approaches (Table 1). Additionally, their ability to alter the immune response to tumor cells is an important consideration when developing immunotherapies against cancer cells. Immunotherapy often targets immune system checkpoints, such as PD-1 and CTL4, with monoclonal antibodies. However, this approach does not reduce the immune cell apoptosis related to high galectin expression in tumor cells. By combining these checkpoint inhibitors with galectin inhibitors, a stronger immune response may be elicited. Table 1 summarizes the current trials of galectin inhibitors which are primarily in mouse models using xenografts of human tumors.

Recent research has also investigated ways in which galectin inhibitors can be used against the mechanisms in which galectins directly modulate tumor development and expansion. Since galectin-1 and galectin-3 are implicated in the increased angiogenesis that drives tumor proliferation and expansion, monoclonal antibodies against these galectins could help mitigate the VEGF pathway and slow cancer progression [47]. Additionally, the inhibition of the VEGF pathway could also be a consideration for treatment and control of ectopic endometrial growth that is associated with endometriosis.

These therapies have shown mixed results in the models, depending primarily on the amount of galectin expression within the tumor cells. Inhibitors have shown promise when used in combination with chemotherapy, as these inhibitors sometimes make the tumor more susceptible to the chemotherapeutic drug. Recombinant galectin-9 infusion has shown effectiveness against malignant cells in vitro and in vivo [46]. It is also worth noting that these inhibitors have not been tested in tumors associated with endometriosis, but studies suggest that galectin inhibitors could be effective against these forms of cancer if they overexpress galectin-1 or galectin-3 [44]. 

## 6. Conclusions

The higher expression levels of galectins measured in patients with endometriosis and associated inflammation suggest that galectins could be used as potential diagnostic biomarkers and/or targets of novel therapeutic approaches for endometriosis. Because of the known involvement of galectins in angiogenesis, cell adhesion, and cellular migration, targeting of specific galectins could be useful in inhibiting the progression of endometriosis. Particularly in advanced stages, endometriosis can progress to certain cancers. This makes galectins a promising target for therapies aimed at controlling and treating inflammation and cancers associated with endometriosis.

## Figures and Tables

**Table 1:** Potential clinical uses of galectins in the treatment of cancers and endometriosis.

Therapy Type	Examples	Targets	Effect
Galectin-1 inhibitors [44]	Thiodigalactoside Anginex OTX008 Davanat	Breast cancer Ovarian cancer Lung cancer Colon cancer Head and neck cancers Melanoma Endometriosis	Induce apoptosis, reduce angiogenesis, restore T cell surveillance, reduce lung metastasis
Galectin-3 inhibitors [44]	G3-C12 Modified Citrus Pectin (MCP), GCS-100	Leukemia Ovarian cancer Prostate cancer Colon cancer Endometriosis	Induce apoptosis, inhibit tumor growth/cell cycle inhibition, increase responsiveness to chemotherapy, increase T cell activity
Recombinant Galectin-9 [46]	hG9NC	Hematological cancer Dermatological cancers Gastrointestinal cancers	Prevent T cell apoptosis Induce apoptosis of malignant cells
